# A cell-free enzymatic activity assay for the evaluation of HIV-1 drug resistance to protease inhibitors

**DOI:** 10.3389/fmicb.2015.01220

**Published:** 2015-10-31

**Authors:** Satoko Matsunaga, Takashi Masaoka, Tatsuya Sawasaki, Ryo Morishita, Yasumasa Iwatani, Masashi Tatsumi, Yaeta Endo, Naoki Yamamoto, Wataru Sugiura, Akihide Ryo

**Affiliations:** ^1^Department of Microbiology, School of Medicine, Yokohama City UniversityYokohama, Japan; ^2^Department of Infection and Immunology, Clinical Research Center, National Hospital Organization Nagoya Medical CenterNagoya, Japan; ^3^Proteo-Science Center, Ehime UniversityMatsuyama, Japan; ^4^CellFree Sciences Co., Ltd.Matsuyama, Japan; ^5^Department of AIDS Research, Nagoya University Graduate School of MedicineNagoya, Japan; ^6^Department of AIDS Research, AIDS Research Center, National Institute of Infectious DiseasesTokyo, Japan; ^7^Department of Microbiology, Yong Loo Lin School of Medicine, National University of SingaporeSingapore, Singapore

**Keywords:** HIV-1, protease, cell-free protein synthesis, cell-free drug susceptibility assay, drug resistance

## Abstract

Due to their high frequency of genomic mutations, human retroviruses often develop resistance to antiretroviral drugs. The emergence of drug-resistant human immunodeficiency virus type 1 (HIV-1) is a significant obstacle to the effective long-term treatment of HIV infection. The development of a rapid and versatile drug-susceptibility assay would enable acquisition of phenotypic information and facilitate determination of the appropriate choice of antiretroviral agents. In this study, we developed a novel *in vitro* method, termed the Cell-free drug susceptibility assay (CFDSA), for monitoring phenotypic information regarding the drug resistance of HIV-1 protease (PR). The CFDSA utilizes a wheat germ cell-free protein production system to synthesize enzymatically active HIV-1 PRs directly from PCR products amplified from HIV-1 molecular clones or clinical isolates in a rapid one-step procedure. Enzymatic activity of PRs can be readily measured by AlphaScreen (Amplified Luminescent Proximity Homogeneous Assay Screen) in the presence or absence of clinically used protease inhibitors (PIs). CFDSA measurement of drug resistance was based on the fold resistance to the half-maximal inhibitory concentration (IC_50_) of various PIs. The CFDSA could serve as a non-infectious, rapid, accessible, and reliable alternative to infectious cell-based phenotypic assays for evaluation of PI-resistant HIV-1.

## Introduction

Mortality associated with human immunodeficiency virus (HIV)-related diseases has declined significantly since 1996, when antiretroviral therapy (ART) regimens were introduced as standard interventions for affected patients. However, these regimens increased the risk of emergence of drug-resistant strains of HIV-1 during long-term management of infected patients (Gupta et al., 2008). Indeed, the high mutation rates and replication capacity of HIV-1 contribute to the generation of drug-resistant variants (Perelson et al., 1996). The emergence of drug resistance significantly impairs the efficacy of ART (Gupta et al., 2008). Hence, drug resistance testing now provides important information when selecting the most appropriate antiretrovirals for treatment of HIV, leading to improved therapeutic outcomes (Haupts et al., 2003; Bansi et al., 2011).

Currently, genotype- and phenotype-based assays are the two major approaches for evaluating drug resistance of HIV-1. Genotypic assays detect mutations that cause drug resistance, and have the advantages of being less expensive and more rapid than phenotypic methods. However, genotypic approaches have several significant disadvantages: they provide only an indirect measure of drug resistance, and they cannot provide a definitive result, especially in the case of viral strains that have accumulated complex genetic mutations (Hanna and D’Aquila, 2001). In addition, the results of genotyping are sometimes discordant between interpretation algorithms (Ravela et al., 2003), and these strategies can only monitor the majority of quasispecies (Derdelinckx et al., 2003). On the other hand, cell culture–based phenotypic assays provide a direct measurement of drug susceptibility and have the significant advantage of being able to measure the cumulative effects of multiple mutations (Robinson et al., 2000). The main disadvantage of a phenotypic assay is the considerable time needed for completion (∼4 weeks) and the requirement for a Bio-Safety Level 3 (BSL-3) laboratory. Moreover, virus isolation or the use of recombinant virus may select for replication-competent viruses in the cell-culture system at the expense of populations of drug-resistant strains that often exhibit lower replication fitness (Nijhuis et al., 2001). Therefore, there is an urgent demand for a rapid and reliable phenotypic assay that would allow easier acquisition of phenotypic information and facilitate understanding of associated genotyping results without the use of cell-culture.

As a step toward resolving these limitations, we developed a novel CFDSA that combines a wheat germ cell-free protein synthesis system (Ogasawara et al., 1999; Madin et al., 2000; Sawasaki et al., 2002) and the Amplified Luminescent Proximity Homogeneous Assay, AlphaScreen (Eglen et al., 2008; Matsunaga et al., 2012). Our method can readily produce full-length active HIV-1 PR proteins from PCR products and measure their enzymatic activity without intervening protein purification. We show here that CFDSA provides an attractive means for analyzing HIV-1 PR drug resistance.

## Materials and Methods

### Reagents

Wheat germ extracts were obtained from CellFree Sciences, Co. (Yokohama, Japan). Anti-HIV-1 PR monoclonal antibody was purchased from Abcam (clone no. 1696, Cambridge, UK). GST antibodies were purchased from GE Healthcare Biosciences (Pittsburgh, PA, USA). Drug-resistant HIV-1 molecular clones were provided by the AIDS Research Center, National Institute of Infectious Diseases, Japan (Takeuchi et al., 2002).

### Construction of *in vitro* Transcription Templates

*In vitro* transcription templates for each HIV-1 PR gene were constructed by split-primer PCR as described previously. To generate transcription templates, the first round of PCR was performed with 10 ng/μl of each plasmid using 100 nM of a target-specific forward primer containing the S1 sequence at the 5′ end (5′-CCACCCACCACCACCAATGTTTTTTAGGGAAGATCTGGCC-3′; underlined nucleotides indicate the S1 sequence (Takai et al., 2010), and non-underlined nucleotides indicate the 5′-coding region of the target gene) and reverse primer 1 (5′-CCTGATATAGGAAGGCCGGATAAGACGCGACCGGCGTCGCATCCGGCGCTAGCCGTAAATTCTATACAAAAACTTATTAGCCATCCATTCCTGGCT-3′). The second round of PCR was performed with 1/100th volume of the first PCR product using 100 nM of primer SPu (5′- GCGTAGCATTTAGGTGACACT-3′; Takai et al., 2010), 100 nM of primer sUTR (5′-ACTACCTGATATAGGAAGGCCG-3′), and 1 nM of primer deSP6E01 (5′- GGTGACACTATAGAACTCACCTATCTCCCCAACACCTAATAACATTCAATCACTCTTTCCACTAACCACCTCCACCCACCACCACCAATG-3′). As a substrate corres ponding to the p2–p7 junction of HIV-1 Gag, the p2/p7-bls (bls: biotin ligase sequence; GLNDIFEAQKIEWHE) fusion gene was inserted into vector pEU-E01-GST-MCS (CellFree Sciences, Yokohama, Japan), and amplified using primers SPu and AODA2303 (5′-GTCAGACCCCGTAGAAAAGA-3′) with ExTaq (Takara Bio). Gag genes derived from HXB2 were amplified by PCR and cloned into vector pEU-E01. Transcription templates for Gag were constructed by PCR following the method described above, using primers SPu and AODA2303.

### *In Vitro* Transcription and Cell-free Protein Synthesis

*In vitro* transcription and cell-free protein synthesis were performed using WEPRO7240 wheat germ extract (CellFree Sciences, Yokohama, Japan). Transcription was performed using SP6 RNA polymerase, as described previously (Matsunaga et al., 2014). The translation reaction was performed in bilayer mode with slight modifications. Briefly, translation mixture (forming the bottom layer) consisted of 10 μl of each mRNA (usually 30–35 μg), 10 μl of WEPRO7240 (CellFree Sciences, Yokohama, Japan), and 0.8 μl of 1 μg/μl creatine kinase (Roche Diagnostics K. K., Tokyo, Japan) in 20.8 μl of SUB-AMIX^®^ (CellFree Sciences, Yokohama, Japan). SUB-AMIX (206 μl) was placed on top of the translation mixture, thus forming the top layer. After incubation at 16°C for 16 h, synthesized proteins were confirmed by immunoblotting. For biotinylation of the substrate, 1 μl (50 ng) of crude biotin ligase (BirA) expressed in a wheat germ cell-free system was added to the bottom layer, and 0.5 μM (final concentration) of D-biotin (Nacalai Tesque, Inc., Kyoto, Japan) was added to both the top and bottom layers, as described previously (Matsuoka et al., 2010). As an initial experimental test, radiolabeled protein was produced by cell-free synthesis to confirm the yield and solubility of generated proteins as described previously (Kamura et al., 2005). In actual drug susceptibility testing, quantitations of synthesized HIV-1 PR proteins were performed by densitometric scanning of Coomassie Brilliant Blue (CBB)-stained bands as compared with purified HIV-1 PR or bovine serum albumin (BSA) (Madin et al., 2000).

### Immunoblotting

Five microliters of cell-free synthesized PRs (equivalent to ∼50 ng) was boiled in 2.5 μL of 3X SDS sample buffer [150 mM Tris-HCl (pH 6.8), 6% SDS, 30% glycerol, and 0.6% bromophenol blue]. After separation by 15% SDS-PAGE, the proteins were electrotransferred onto a PVDF membrane (Bio-Rad, Hercules, CA, USA) by application of 100 V for 1 h. The membrane was then blocked in Tris-buffered saline (TBS) containing 5% (w/v) skim milk for 1 h, and then incubated for 1 h with a HIV PR antibody (ab8327; Abcam, Cambridge, MA, USA) in TBS containing 0.1% (v/v) Tween 20 (TBST; 1:1000 dilution) as described previously (Matsunaga et al., 2014). After three washes with TBST, the filter was incubated for 40 min in PBS containing goat-anti mouse IgG-HRP antibody (1:5000; GE Healthcare). After an additional three washes in TBST, HIV PR proteins were detected with SuperSignal West Dura Extended Duration Substrate (Thermo Fisher Scientific, Rockford, IL, USA) on a Lumi-Imager F1 (Roche Diagnostics GmbH, Mannheim, Germany).

### Cell-free Enzymatic Assay using a Luminescence Detection System

CFDSA was performed using cell-free–synthesized PR, substrate peptide, and the AlphaScreen^®^ system (PerkinElmer, Boston, MA, USA). Briefly, to bind PR and PI, pre-incubation was performed in a total volume of 10 μl containing 3 μl of cell-free–synthesized PR (∼0.25 μM as a homodimer) and 1X SUB-AMIX^®^ in the presence of serially diluted PI (0.1 to 10^5^ nM indinavir, ATV, APV, or DRV). The mixture was incubated at 37°C for 30 min in a 384-well Alphaplate (PerkinElmer). The enzymatic reaction was initiated by the addition of 5 μl of 0.25 μM cell-free–synthesized GST-p2/p7-bls substrate to each well, followed by incubation at 37°C for 2 h. Detection of PR activity was performed essentially as described in the AlphaScreen IgG detection kit instruction manual (PerkinElmer). Briefly, 10 μl of detection mixture containing 20 mM Tris-HCl (pH 7.5), 0.2 mM DTT, 5 mM MgCl_2_, 5 μg/ml anti-GST antibody, 1 mg/ml BSA, 0.1 μl streptavidin-coated donor beads, and 0.1 μl anti-IgG acceptor beads were added to each well of the 384-well plate, followed by incubation at 23°C for 1 h in the dark. Luminescence signals were analyzed with the AlphaScreen detection program using the EnSpire software (PerkinElmer), and light intensities in the presence of the serially diluted inhibitors were used to calculate their IC_50_ values. PI-resistance levels were determined by comparing the IC_50_ values of drug-resistant PR with those of NL4-3 PR. The results for each PR were normalized based on protein productivity, as determined by liquid scintillation counting. IC_50_ values were calculated using XLfit (ID BUSINESS SOLUTIONS, Guildford, UK). The criteria for CFDSA measurement of resistance were as follows: fold resistance value (FRV) = IC_50_ ratio of test PR/IC_50_ ratio of WT PR.

### Clinical Isolates

Serum samples were collected from AIDS patients diagnosed at Nagoya Medical Center. This study was conducted according to the principles expressed in the Declaration of Helsinki, and was approved by the Institutional Review Boards of the National Institute of Infectious Diseases (approval number: 166) and Nagoya Medical Center (approval number: 2010-310). All patients provided written informed consent for collection of samples and subsequent analysis. Some viral clones containing common clusters of drug-resistant mutations were provided by Dr. Robert W. Shafer at Stanford University.

## Results

### Synthesis of Enzymatically Active HIV-1 Protease using a Wheat Germ Cell-free System

**FIGURE 1:**
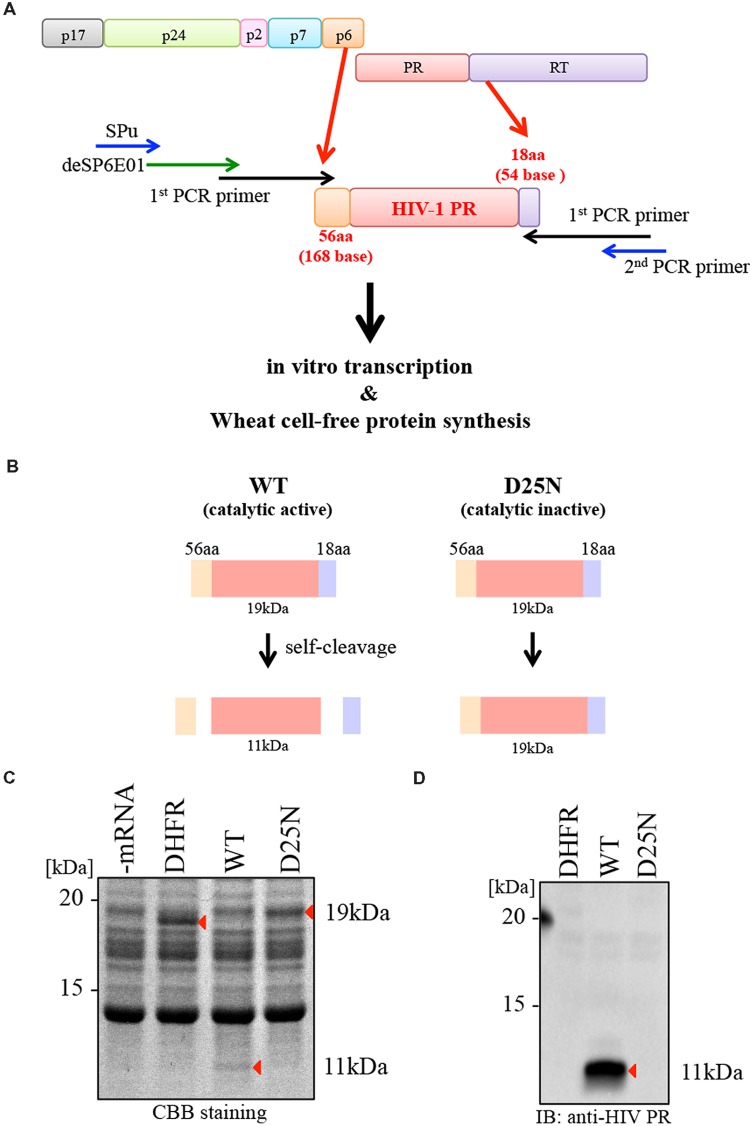
**Synthesis of catalytically active HIV-1 PR using the wheat germ cell-free system. (A)** Schematic representation of rapid production of HIV-1 PR using a wheat germ cell-free system. A transcriptional template, including the HIV-1 PR open reading frame flanked by N-terminal 168 bases (56 aa) and C-terminal 54 bases (18 aa), produced by split-primer PCR as described in section “Materials and Methods.” Cell-free translation was carried out using products of *in vitro* transcription. **(B)** The formation of WT PR (catalytic active) or D25N PR (catalytic inactive) after translation. WT PR generated as a 11 kDa protein by self-cleavage. D25N PR (catalytic inactive) generated as a 19 kDa protein lacking self-cleavage activity. **(C,D)** WT or D25N PRs (-mRNA and DHFR as a negative control) were separated by SDS-PAGE followed by CBB-staining **(C)** and immunoblotting using anti-HIV-1 PR antibody that recognizes only mature HIV protease (PR) but not its precursor **(D)**. Arrows depict protein products.

To synthesize catalytically active HIV-1 PR, we initially generated a transcriptional template of this enzyme by PCR, using the HIV-1_NL4-3_ clone as a wild-type (WT) reference sample. We designed a transcription template encoding the open reading frame of HIV-1 PR flanked by 56 N-terminal amino acids (Gag p6 region) and 18 C-terminal amino acids (the reverse-transcriptase region), as shown in **Figure 1A**. The *in vitro* transcription template for the HIV-1 PR gene was constructed by split-primer PCR using primers encoding the SP6 and E01 sites, as described in section “Materials and Methods.” To generate a catalysis-incompetent PR, we designed a PR mutant harboring the catalytic active site substitution D25N (D25N; **Figure 1B**). All cDNA templates were subjected to cell-free transcription/translation and then separated by SDS-PAGE. By CBB staining, WT PR migrated at 11 kDa (as the truncated form of PR) due to self-cleavage of the flanking 56 and 18 amino acids at the N- and C-terminal ends, respectively, whereas D25N PR migrated at 19 kDa, corresponding to full-length PR with the flanking sequences (**Figures 1C,D**). Immunoblot analysis with anti-HIV-1 PR antibody recognizing mature form of HIV PR, only detected the cleaved form of WT PR at the expected size (∼11 kDa; **Figure 1D**).

### Measurement of HIV-1 Protease Activity using AlphaScreen

**FIGURE 2:**
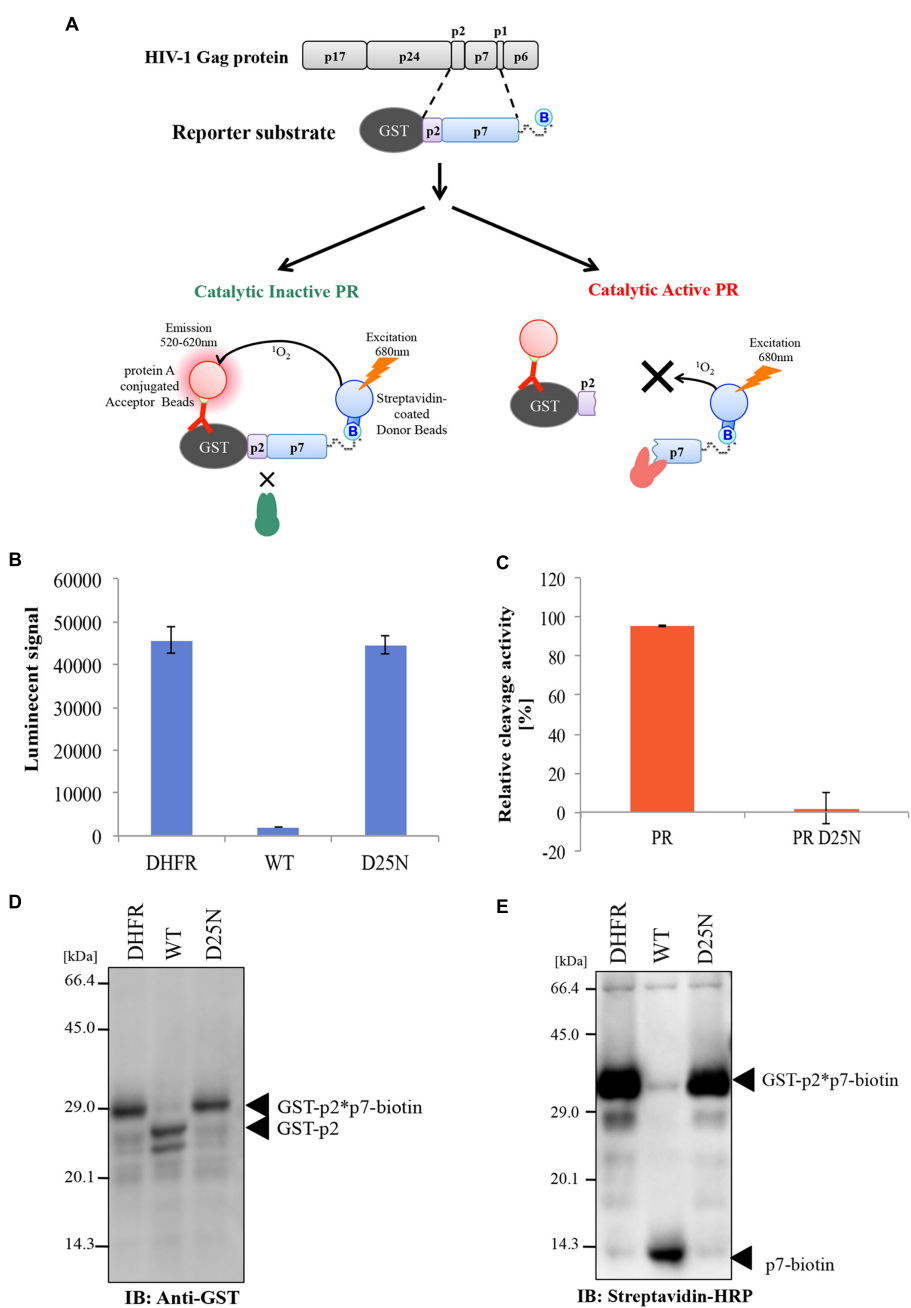
**Measurement of HIV-1 PR cleavage activity using AlphaScreen. (A)** Design of a reporter substrate (GST-p2/p7-biotin) used in the assay. Schematic representation of the CFDSA used to evaluate drug susceptibility of HIV-1 PR. Cell-free–synthesized PR was incubated with the reporter substrate. Subsequently, protein A–conjugated acceptor beads and streptavidin-coated donor beads were added and bound to the substrate. In this system, when PR does not cleave the test substrate, energy is converted from the donor beads to acceptor beads, resulting in light emission at 520–620 nm (right upper part). By contrast, when PR cleaves the substrate, no light is produced (right lower part). **(B,C)** Cleavage activity of HIV-1 PR was quantitated by AlphaScreen as shown in **(A)**. Luminescent AlphaScreen signal **(B)** and relative enzymatic activity **(C)** are listed. Each bar represents the mean ± SD of four independent experiments. **(D,E)** Conformation of cleavage of the tester polypeptide by immunoblot analysis. The reporter substrate was incubated with HIV-1 PR or D25N mutant, and the reaction mixtures were then separated by SDS-PAGE. Substrate cleavage was analyzed by immunoblotting against a GST antibody (left) or streptavidin conjugated with peroxidase (right). Arrows depict protein products.

For the quantitative and high-throughput measurement of HIV-1 PR activity using AlphaScreen technology, we designed a reporter substrate comprising a partial Gag p2-p7 junction peptide flanked by N-terminal GST and C-terminal biotin binding sequence (GST-p2/p7-biotin) as described in **Figure 2A**. Using the reporter substrate, we attempted to measure the cleavage activity of HIV-1 PR by AlphaScreen (Matsunaga et al., 2012). Cell-free–synthesized HIV-1 PRs were incubated with the reporter substrate, followed by the addition of AlphaScreen streptavidin donor and protein A acceptor beads with anti-GST antibody (**Figure 2A**). In this system, when PR does not cleave the reporter substrate, singlet oxygen energy can be transmitted from the donor beads to the acceptor beads, resulting in emission of light. By contrast, when PR cleaves the substrate, no light is produced.

Consistent with the theory described above, WT HIV-1 PR, but neither D25N PR nor DHFR (used as a negative control), diminished the AlphaScreen luminescence signal, indicating proteolytic cleavage of the reporter polypeptide (**Figure 2B**). The cleavage activity of PR was normalized against the luminescence activity of DHFR (**Figure 2C**). Parallel immunoblot analysis with an anti-GST antibody or streptavidin-HRP demonstrated that WT PR, but not D25N PR, efficiently digested the reporter substrate into the expected cleavage products (GST -p2 and p7-biotin; **Figures 2D,E**).

### Evaluation of Drug-resistant HIV-1 PR by CFDSA

**FIGURE 3:**
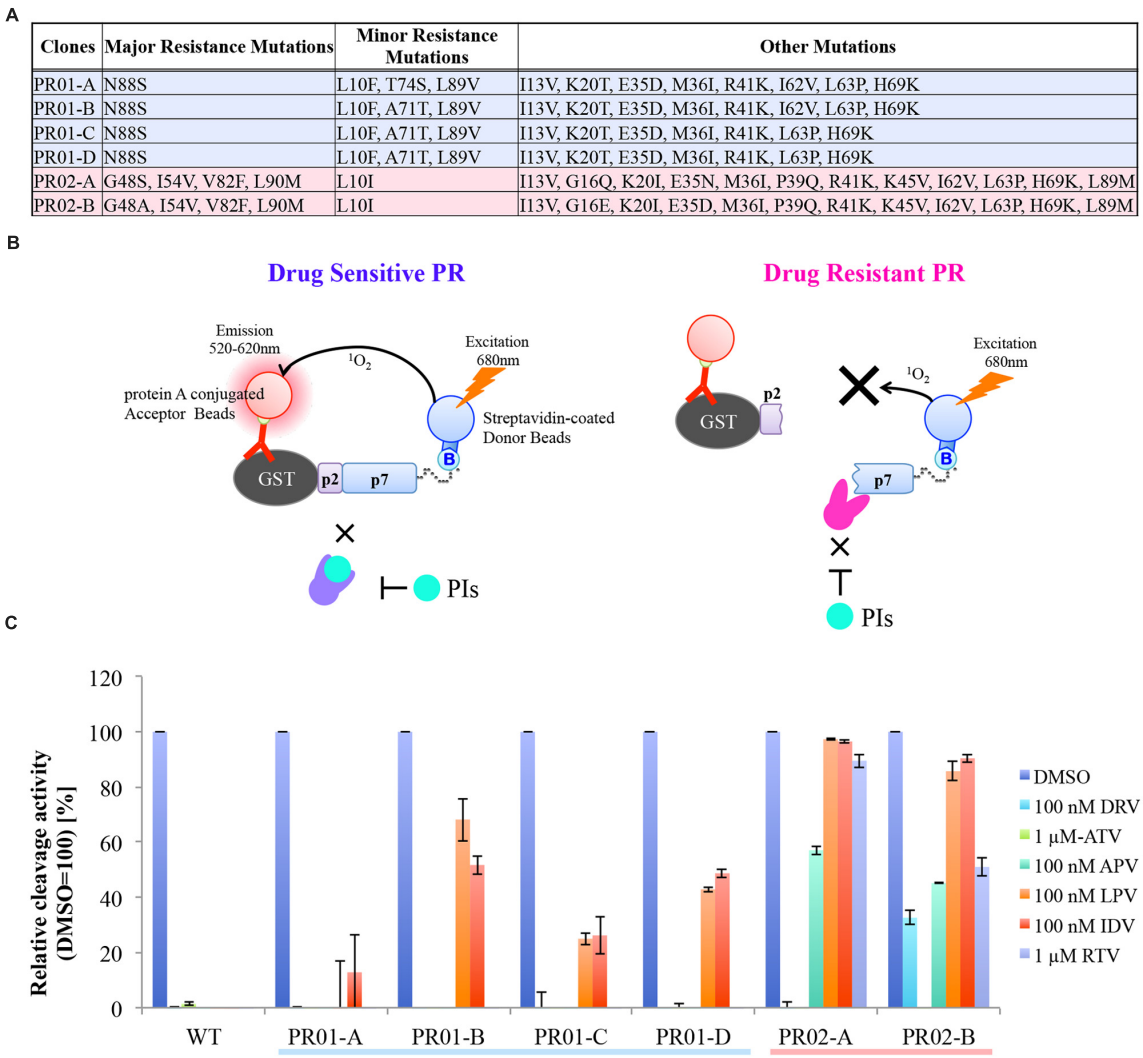
**HIV-1 PR drug resistance profiles, as determined by CFDSA in a single-concentration experiment. (A)** List of six PRs from clinically drug-resistant clones used in this assay. **(B)** Schematic representation of CFDSA in case of drug sensitive PR or drug resistant PR. Drug sensitive PR does not cleave the reporter substrate with PIs, energy is converted from the donor beads to acceptor beads, resulting in light emission at 520–620 nm. By contrast, when drug resistant PR cleaves the substrate regard less with PIs, no light is produced. **(C)** WT PR and six patient-derived drug-resistant PR mutants were pre-incubated with the indicated protease inhibitors (PI; DRV/darunavir, 100 nM; APV/amprenavir, 100 nM; ATV/atazanavir, 1 mM; IDV/indinavir, 100 nM; LPV/lopinavir, 100 nM; and RTV/ritonavir, 1 mM), and then subjected to CFDSA. Relative cleavage activities were listed. Each bar represents the mean ± SD of two independent experiments.

We next investigated whether our assay system is applicable to drug-resistance testing for HIV-1 PR. As an initial approach, we examined the susceptibility of WT HIV-1 PR, or six PRs from clinically drug-resistant clonal isolates (**Figure 3A**), to six different PIs (DRV; APV; ATV; IDV; LPV, lopinavir; and RTV, ritonavir) at a single effective concentration. Although all HIV-1 PIs tested markedly inhibited WT HIV-1 PR, HIV-1 PRs derived from drug-resistant clones exhibited significantly variable drug resistance at the indicated concentrations (**Figures 3B,C**). Notably, PRs with the comparable “major” or “minor” mutations exhibited distinct drug-resistance profiles, indicating the advantage of CFDSA over the conventional genotyping methods that target only these mutations (**Figures 3B,C**).

**FIGURE 4:**
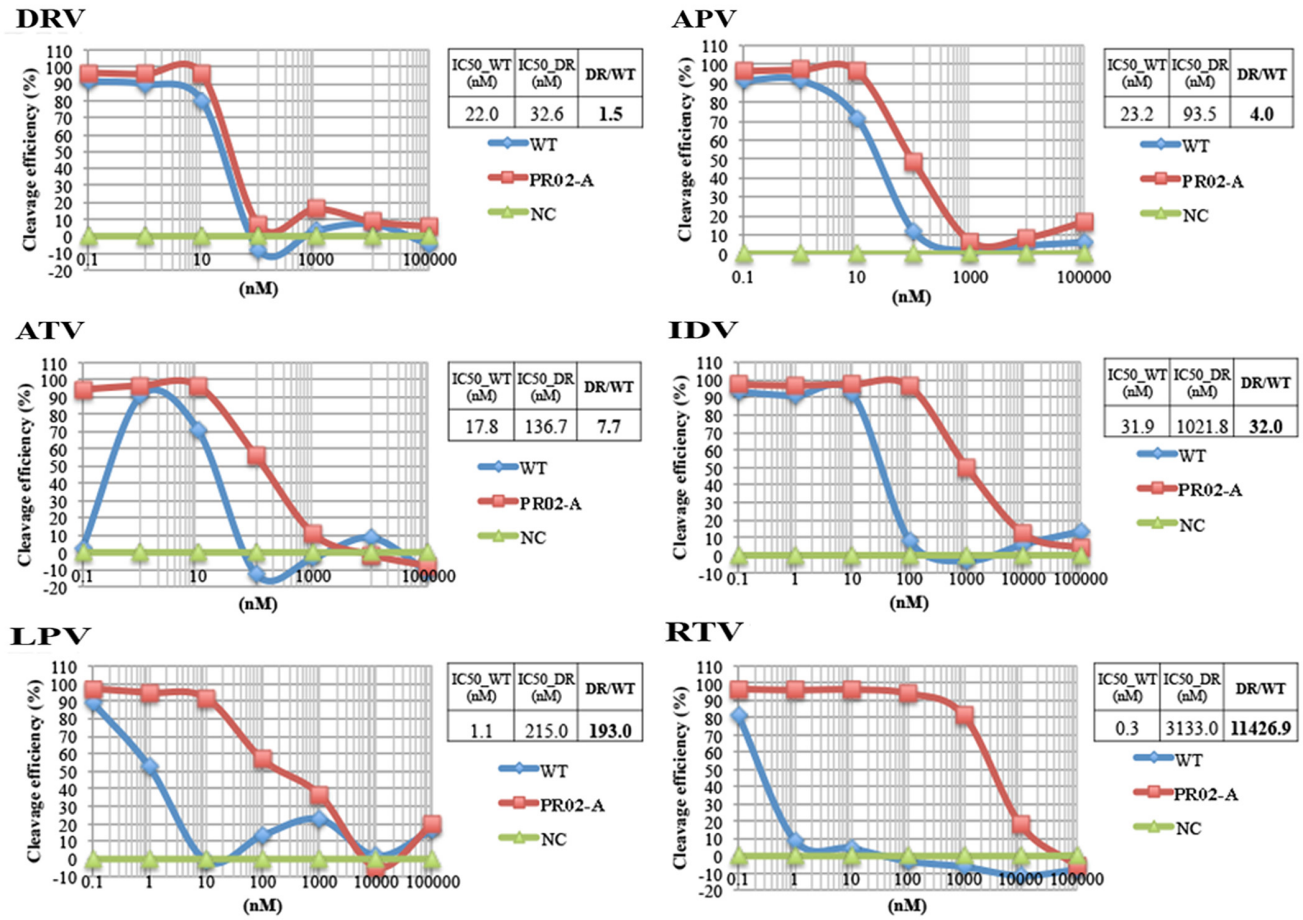
**Dose-response curve of HIV-1 PR activity in the presence of PIs, as determined by CFDSA.** WT HIV-1 PR or a drug-resistant PR (PR02-A, as shown in **Figure 3**) harboring L10I/G48S/I54V/V82F/L90M mutations was tested in the presence of the indicated concentration of PIs by CFDSA. Luminescent AlphaScreen signals were plotted for each PI, and IC_50_ values were calculated using XLfit for each inhibitor. Fold resistance value (FRV) was calculated using the IC_50_ value of the drug-resistant PR divided by the IC_50_ value of the WT PR. NC, negative control (DHFR).

We next attempted to determine IC_50_ values by titrating the PIs (**Figure 4**). WT HIV-1 PR and a drug-resistant PR harboring the L10I/G48S/I54V/V82F/L90M mutations were tested. For WT PR, the IC_50_ values for DRV, APV, ATV, IDV, LPV, and RTV were 22.0, 23.2, 17.8, 31.9, 1.1, and 0.3 nM, respectively. For the mutant PR, the IC_50_ values (fold-resistance to WT PR) were 32.6 (1.5-fold higher), 93.5 (4.0-fold higher), 136.7 (7.7-fold higher), 1021.8 (32.0-fold higher), 215.0 (193.0-fold higher), and 3133.0 (11426.9-fold higher) nM, respectively (**Figure 4**). These results indicate that our current assay system can readily determine the susceptibility or resistance of a mutated PR to a particular drug based on the fold change in IC_50_ values relative to those of WT PR.

### Comparison of CFDSA with PhenoSense Assay

**FIGURE 5:**
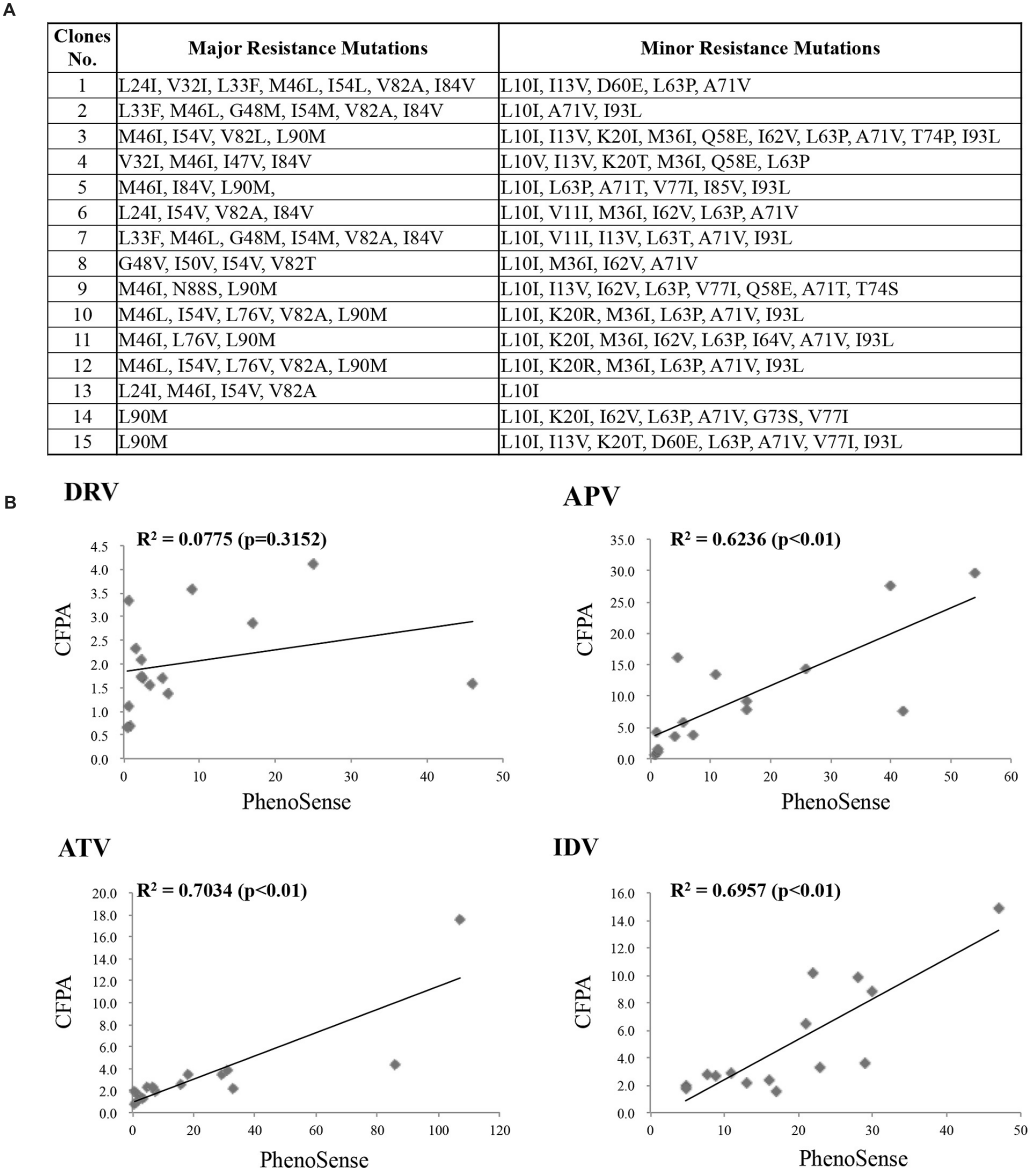
**Comparison of CFDSA with the PhenoSense assay. (A)** Information about the PR mutants used in this assay. **(B)** Assessment of drug susceptibility and resistance determined by CFDSA, compared to those determined by the PhenoSense^®^ assay. The y-axis indicates CFDSA drug-resistant scores, whereas the x-axis represents PhenoSense^®^ scores.

To evaluate the practical potential of our assay, we compared drug susceptibility profiles obtained via CFDSA with those determined by the phenotypic technique, PhenoSense^®^. For this purpose, we synthesized 15 patient-derived drug resistant PRs with multiple mutations and measured their IC_50_ values against the same four PIs (DRV, APV, ATV, and IDV). To minimize the effects of cleavage-site mutations, we used molecular clones in which mutant PR genes were inserted into the wild-type pNL4-3 clone. The mutants’ sequence profiles are summarized in the lower panel of **Figure 5A**.

Drug-resistance scores determined by CFDSA and PhenoSense^®^ were plotted in scatter plots. The CFDSA results were significantly positively correlated with those of PhenoSense^®^ for ATV, APV, and IDV (**Figure 5B**); for DRV, no correlation was found probably due to its mechanism of action (**Figure 5B**; see discussion). These results indicate that PI-resistance results obtained by CFDSA are almost, but not completely, consistent with those determined by conventional cell-based drug-susceptibility assays.

## Discussion

The phenotypic drug-resistance assays in current use are complex, labor-intensive, and expensive. Therefore, there is an urgent need for a simpler, safer, and less expensive method for drug resistance testing (Garcia-Lerma and Heneine, 2002). Here, we describe the development of a novel *in vitro* enzymatic activity assay that provides a rapid and reliable method for evaluating the PI-resistance of clinical HIV samples. Our method is based on the direct measurement of the catalytic activity of HIV-1 PR synthesized using a wheat cell-free system.

The advantages of CFDSA in comparison with conventional cell-based phenotypic assays include (1) a relatively lower cost than alternative *in vitro* and *in vivo* screening technologies (∼50–60 cents/well including protein synthesis); (2) greater rapidity than cell-based phenotypic assays (the entire test procedure is completed in 2 or 3 days vs. several weeks); (3) no requirement for a Bio-Safety Level 3 (BSL3) containment; (4) has the versatility to be used in high-throughput assays with multi-well plates; and (5) is a “mix and measure” assay system which can potentially be automated. Although we demonstrate here that our newly developed CFDSA could be a potentially powerful tool for the measurement of catalytic activity of HIV-1 PR, it is still immature to use for practical and actual HIV-1 PR drug-resistance testing in clinics. Further studies involving a much larger sample size with multiple drug-resistant mutations will be necessary to determine whether CFDSA is truly applicable to clinical testing or use as an effective diagnostic tool in the treatment of HIV-1-infected patients.

Several related biochemical methods have been used to evaluate HIV-1 PI susceptibility (Yu et al., 1995; Gutierrez et al., 2002; Hoffmann et al., 2005; Hu et al., 2005; Matsuda et al., 2007). The basic principle involved in these procedures is to incubate the recombinant PR, substrate peptide, and PI *in vitro*, and then measure the amount of substrate cleaved by PR. The advantage of this approach is that it directly evaluates the drug-resistance phenotype based on the catalytic activity of PR, even in cases when there are cumulative effects of a large number of mutations. However, it is often difficult to produce sufficient quantities of enzymatically active PR in conventional cell-based protein expression systems, such as *Escherichia coli* (*E. coli*). In *E. coli*, HIV PR is usually expressed in the inclusion body fraction due to its insolubility and cytotoxicity (Cheng et al., 2006). In comparison to cell-mediated procedures, the wheat germ cell-free system is advantageous for the efficient preparation of high-quality proteins with natural folding and high enzymatic activity, both of which enable high-throughput functional assays (Endo and Sawasaki, 2006). Moreover, the wheat germ system is suitable for the generation of toxic viral proteins such as HIV-1 PR.

Cell-free drug susceptibility assay was designed to evaluate the drug-resistance properties of PR enzymes that harbor complex and multiple mutations, a key limitation of genotypic assays (Baldanti et al., 2004). Assessment of drug resistance of HIV PRs that harbor highly complex mutations, or PRs derived from non-B subtypes of the virus, is currently the focus of a great deal of attention (Llibre et al., 2010). However, the aforementioned limitations of genotypic assays, which can only indirectly evaluate this characteristic, may result in a lack of information. In this regard, when scoring drug susceptibility, genotypic assays usually consider only predetermined major and minor drug-resistance mutations. On the other hand, our CFDSA method can evaluate PI resistance based on actual enzymatic activity in real time. Thus, CFDSA provides a means for predicting the drug resistance of PRs that have accumulated complex mutations.

Our results demonstrate that the drug-resistance scores of CFDSA were highly concordant with those of PhenoSense^®^. However, there were some discrepancies, specifically in regard to susceptibility to DRV. DRV is a potent antiretroviral drug that can block the dimerization of HIV-1 PR, and also has high affinity for the mature enzyme, although the mechanism by which it inhibits dimerization has not been well characterized (Huang and Caflisch, 2012). Because CFDSA utilizes mature HIV-1 PR to directly evaluate enzymatic activity, and does not reflect differences in maturation, the results obtained for DRV may not accurately represent the situation *in vivo*. Further biochemical analysis will be necessary to clarify these intriguing findings.

In summary, we show here that our method provides a simple and biologically relevant means for quantitative evaluation of drug-resistant HIV-1 PRs using existing therapeutics.

## Author Contributions

SM and TM designed and performed the research, analyzed the data, and wrote the manuscript; TS and RM performed the research, developed the screening system and analyzed the data; NY analyzed the data and edited the manuscript; YI, MT, and YE contributed reagents and analyzed the data; WS and AR directed the research, analyzed the data, and wrote the manuscript.

## Conflict of Interest Statement

The authors declare that the research was conducted in the absence of any commercial or financial relationships that could be construed as a potential conflict of interest.

## Abbreviations

APV: amprenavir
ATV: atazanavir
CFDSA: Cell-free drug susceptibility assay
DHFR: dihydrofolate reductase
DRV: darunavir
GSS: genotypic susceptibility score
GST: glutathione *S*-transferase
HAART: highly active antiretroviral therapy
HIV-1: human immunodeficiency virus type 1
IDV: indinavir
PI: protease inhibitor
PR: protease
